# An update on the application of nano-scaled carriers against fluconazole-resistant *Candida *species: nanostructured lipid carriers or solid lipid nanoparticles? 

**DOI:** 10.18502/cmm.5.4.1965

**Published:** 2019

**Authors:** Maryam Moazeni, Majid Saeedi, Hamidreza Kelidari, Mojtaba Nabili, Amirhossein Davari

**Affiliations:** 1 Invasive Fungi Research Center, Mazandaran University of Medical Sciences, Sari, Iran; 2 Department of Medical Mycology, School of Medicine, Mazandaran University of Medical Sciences, Sari, Iran; 3 Department of Pharmaceutics, Mazandaran University of Medical Sciences, Sari, Iran; 4 Pharmaceutical Sciences Research Center, Mazandaran University of Medical Sciences, Sari, Iran; 5 Department of Medical Laboratory Sciences, Sari Branch, Islamic Azad University, Sari, Iran; 6 Student Research Committee, Mazandaran University of Medical Sciences, Sari, Iran

**Keywords:** Candida, Fluconazole-resistant, NLC, SLN

## Abstract

**Background and Purpose:**

Encapsulation can lead to improved efficacy and safety of antifungal compounds. The attention of scientists has recently turned to biocompatible lipids as the carriers for the delivery of antifungal drugs, such as fluconazole. Although several research reports have already been published on fluconazole loaded solid lipid nanoparticles (FLZ-SLNs) and fluconazole loaded nanostructured lipid carriers (FLZ-NLCs), the possible advantages of NLCs over SLNs have not yet been fully established. Studies performed so far have given several contradictory results.

**Materials and Methods:**

Both formulations of fluconazole were synthesized using probe ultrasonication method and the characteristics were analyzed. Antifungal susceptibility testing (AFST) was performed with FLZ, FLZ-SLNs, and FLZ-NLCs using CLSI document M60 against some common fluconazole-resistant Candida species.

**Results:**

A significant decrease was observed in minimum inhibitory concentration values when both formulations were applied. Nonetheless, FLZ-NLCs were significantly more effective (*P<0.05*). However, three species groups were not statistically different in terms of the activity of FLZ-NLCs.

**Conclusion:**

Based on the obtained results, FLZ-NLCs could reverse the azole-resistance phenomenon in the most common *Candida* species more effectively, as compared to FLZ-SLNs.

## Introduction

Several classes of antifungal drugs have been developed for the treatment of both systemic and superficial fungal infections [1], among which fluconazole (FLZ) is a synthetic fluorinated bis-triazole derivative extensively used owing to its highly effective antifungal activity and its wide antimycotic spectrum. FLZ has notable advantages over other azole drugs, such as better water solubility (of 8 mg/mL, at 37°C), and higher bioavailability. However, systemic therapy with FLZ poses two major problems, including common adverse effects, such as nausea, vomiting, bloating, diarrhea, and abdominal pain, that reduce the patient's compliance to treatment in long-term therapies, and various drug interactions

[ 3 - 5]. FLZ has a relatively large molecular size and hydrophobicity which improves its bioavailability via intravenous or oral routes; however, these characteristics present some problems for the implementation of FLZ in a topical application. Nonetheless, FLZ is rapidly and highly accumulated in the stratum corneum following systemic administration [5]. Owing to this characteristic, FLZ is considered a suitable candidate for topical delivery which could be an effective alternative route in the treatment of superficial fungal infections [2]. However, its widespread use has led to an increase in FLZ- resistant *Candida *strains [6] with intrinsic and/or acquired resistance to FLZ (e.g.,* C. glabrata *and *C. krusei*) [7]. Administration of large doses of the drug and application of multiple therapeutic agents is essential in these cases.

Encapsulation can lead to improved efficacy and safety of this antifungal compound [8]. This technology offers several advantages, such as the protection of active agents, controlled and sustained release, increase in therapeutic effect, and reduction of side effects [9]. In this regard, several antifungal agents have been encapsulated in nanoparticles (NPs) with positive results in animal models and clinical therapy [10, 11].

The attention of the formulation scientists has recently turned to biocompatible lipids as carriers for the delivery of poorly soluble drugs [12], among which lipid nanoparticle-based on solid matrix has gained considerable interest. In general, there exist two types of lipid nanoparticles with solid matrix, namely solid lipid nanoparticles (SLNs) and nanostructured lipid carriers (NLCs) [13]. The SLNs are prepared using solid lipids (i.e., lipids that are solid at room temperature, as well as at body temperature). Theses lipids are biocompatible and biodegradable with generally recognized as safe (GRAS) status. SLNs are proved highly beneficial in a number of ways, such as the use of organic solvents, bioavailability of highly lipophilic, sustained drug release from the nanoparticle matrix, and penetration through skin or mucus barrier due to nano-size. Nonetheless, the amount of encapsulated drug and drug release profile of SLNs may change with storage time. NLCs have been developed as an alternative drug carrier systems as the result of further improvement and reduction of these drawbacks of SLNs. NLC matrix consists of spatially different lipid molecules mixture and normal mixture of solid and liquid lipid which makes more imperfection in the matrix to accommodate more drug molecules, as compared to SLN. Although several research reports have already been published on SLNs and NLCs, the advantages of NLCs over SLNs have not been fully established yet and the studies performed so far have given several contradictory results in this regard[14 - 16]. Concerning this, the aim of the present study was to compare the efficacy of SLNs-FLZ and NLCs-FLZ on the common FLZ-resistant strains of *Candida* species.

## Materials and Methods

***Materials***

Fluconazole (FLZ, Pharmaceutica grade) was obtained from Arasto Pharmaceuticals Chemicals Inc. (Tehran-Iran). Compritol1 888 ATO (CO), Lipocire and Precirol1 ATO 5 were supplied from Gattefossé (Saint-Priest, Cedex, France). Sabouraud dextrose agar (SDA), RPMI medium, stearic acid (SA), Oleic acid, Tween 80 (Tn80), Span 60 (Sn60) and Span 80 (Sn80) were purchased from Merck Co. (Germany). HPLC grade acetonitrile and methanol were provided by the Merck (Germany). Morpholinepropanesulfonic acid (MOPS) was purchased from Sigma Chemical Co., St. Louis, MO (USA). Deionized water was purified using a Milli-Q system (Millipore, Direct-Q). All the other reagents and solvents were either of analytical or high-performance liquid chromatography (HPLC) grades.

***Preparation of fluconazole loaded Solid Lipid Nanoparticles and fluconazole loaded nanostructured lipid carriers***

FLZ-SLNs were prepared using the probe ultrasonication method which had previously been used for the production of lipid nanoparticles [17]. Stearic acid (SA) was selected for the production of both NLCs and SLNs, based on previous studies. The method for FLZ-NLCs preparation was driven from the previously reported studies in the literature since no drug crystals were observed when FLZ and stearic acid were heated together [18]. The NLCs were prepared by melting carrier lipid in its solid form (SA) at 85 °C along with liquid lipid (oleic acid) and a lipophilic surfactant (Span 80) (supplied by Merck, Germany). The molten lipid phase was dispersed in 1/3 of the aqueous solution of hydrophilic surfactant prepared by weighing out 0.84% w/w Tween 80 at the same temperature and sonicated using a probe sonicator (Bandelinsonopuls, Berlin, Germany) for 5 min (Model HD 3200, Prob TT25, 50% power and 14.28 KJ, continuous) to form a pre-emulsion. Conversely, FLZ-SLNs were prepared using SA as solid lipid carrier and a lipophilic surfactant (Span 80) [18]. In brief, 2 g solid lipids with 0.5 g of drug and 0.25 g span 80 (supplied by Merck, Germany) were melted at 85 °C. The hot lipid phase was dispersed in 1/3 aqueous solution containing 0.5 g of hydrophilic surfactant Tween 80 (Tn80) (supplied by Merck, Germany), heated at the same temperature and sonicated by a probe sonicator (Bandelinsonopuls, Berlin, Germany) for 5min to form a coarse pre-emulsion. The mixture was dispersed into 2/3 of an ice-cold surfactant solution kept in an ice-cold bath upon the completion of sonication. The final mixture was sonicated again for 10 min while immersed in the ice bath. This cooling step promoted the formation of the lipid nanoparticles.

***Characterization of nanoparticles***

***Morphology measurement***

 The morphology of drug particles was visualized by field emission scanning electron microscope (FESEM, HITACHI S-4160, and the U.S.A). A drop of suspension was placed on a double-sided carbon tape and dried at 25°C for 24 h for the prepared particles. Prior to visualization, the particles were sputter-coated with gold for 40 s. Transmission electron microscopy (accelerating voltage 100 kV; TEM, CM 30, Phillips, The Netherlands) was used to determine the shape of nanoparticles. Initially, the NLC samples were diluted twice with distilled water. One drop of the diluted sample was placed on a 200-mesh carbon-coated copper grid, stained with 2% phosphotungstic acid solution and dried at room temperature. Representative images of the sample were reported.

***Particle size and zeta potential***

Photon correlation spectroscopy (PCS) with a Malvern Zetasizer ZS (Nano ZA, Malvern Instruments, UK) was applied to determine the particle size and profile the size distribution (polydispersity index, PDI) and zeta potential of the nanoparticles. In this method, the sample was measured at 25°C with an angle detection of 90_. The concentration of the samples for analysis on the Zetasizer was 20–400 kilocounts per second (KCPS) and the intensity of diffraction was 100,000 counts per second. The average particle size and zeta potential of both SLNs and NLCs were measured at 25 °C and a fixed angle of 90° using Zeta SizerNano ZS (Malvern Instruments, UK). The results which are the means of three determinations are depicted as the mean±standard deviation.

***Determination of fluconazole entrapment efficiency***

Entrapment efficiency (EE%) was determined to assess the extent of FLZ incorporation in the nanoparticles by measuring the concentration of the free unloaded FLZ in the aqueous phase of the nanoparticle suspension. To determine the EE% of FLZ in the NLCs and SLNs format, the nanoparticles were subjected to centrifugation for 20 min at 25,000 rpm (HERMLE, Z36HK, Germany) and filtered (pore size: 0.22 mm). The amount of drug in the supernatant was established by HPLC and the experiment was conducted in triplicate. The EE% of the drug was calculated using the following equation: EE%=Wi−f /Wi ×100, where Wi and Wf are the amount of drug added in the formulation and amount of drug in the supernatant, respectively.

***Antifungal susceptibility testing***

***Isolates***

Apart from standard strains of *C. albicans *(ATCC 14053), *C. glabrata *(ATCC 90030) and *C. parapsilosis*, we applied FLZ-resistant strains of *C. albicans*, *Candida parapsilosis sensu stricto* and *C. glabrata *(ATCC22019) to assess the effectiveness of nanoparticles loaded with FLZ as a means of protecting the drug binding sites. The isolates were identified to the species level by a PCR-RFLP method using an *Msp*1 restriction enzyme. Moreover, resistant strains were proven to have MIC≥ 8 μg/ml for *C. albicans / C. parapsilosis* and MIC ≥ 64 μg/ml for *C. glabrata* against FLZ (data not shown) using standard protocol CLSI documents M27-A3 and M27-S4. Stock cultures were initially grown on yeast extract peptone dextrose agar (YEPD; 1% yeast extract, 2% Bacto Peptone, 2% dextrose) at 35°C for a period of 48 hours to obtain fresh viable yeast cells. To perform antifungal susceptibility testing, the isolates were inoculated onto Sabouraud dextrose agar (SDA; supplied by Merck, Germany) and incubated at 35°C for 24 h.

***Antifungal agents***

Antifungal susceptibility testing (AFST) was performed with FLZ, FLZ-SLNs and FLZ-NLCs. FLZ was dissolved in sterile water, and a two-fold dilution was prepared in RPMI 1640medium (with L-glutamine, without bicarbonate) and buffered to pH 7.0 using a 0.165M solution of MOPS (3-N-Morpholinepropanesulfonic acid which is an excellent buffer form any biological systems at near-neutral pH was purchased from Sigma Chemical Co., St. Louis, MO). Two-fold dilutions of both FLZ-SLNs and FLZ-NLCs were synthesized, as well. Notably, the dilutions were prepared with equal concentrations of FLZ i.e. 16- 0.0125 µg/ml of FLZ-SLNs and FLZ-NLCs were applied. New formulations were freshly synthesized and applied for AFST one week later.

***Antifungal susceptibility testing***

Recommendations of CLSI M59 and M60 documents determined a minimum inhibitory concentration (MIC) against both FLZ and FLZ-NLCs[19, 20]. FLZ and FLZ-NLCs were dispensed into 96-well microdilution trays at a final concentration of 0.063–64 µg/ml and 0.1–8% (0.1–8 g/ml), respectively. *Candida* blastospore suspensions were prepared from isolates grown for 24 h. Yeast colonies were suspended in sterile saline solution and spectrophotometrically adjusted at 530 nm to optical densities (OD) from 0.09-0.13 (absorbance at 530 nm within a range of 0.09-0.13 is equal to the transmission of 75-77%). The final size of the stock inoculums falls within the range of 1×10^6^-5×10^6^ CFU/ml. A working suspension was then made using a 1:10 dilution followed by a 1:100 dilution of the stock suspension with RPMI medium which resulted in two times test inoculums (1×10^3^-5×10^3^ CFU/ml). Microdilution plates were incubated at 35°C and examined visually after 24 and 28 h as the concentration of drug that elicited significant inhibition (approximately 50%) of growth, as compared to a drug-free control. Due to its self-turbidity, an inverted microscope (Motic AE31, Hong Kong, China) was used for the examination of the results in the case of FLZ-loaded NLCs. *C. parapsilosis* (ATCC 22019) was selected as the quality control to be used with each new series of MIC plates.

***Statistical analysis***

Data were expressed as mean±standard deviation. Analysis of variance (ANOVA) was used following Dunnett’s test. Mann-Whitney U test was applied to determine the effects of FLZ-NLCs and FLZ-SLNs on the reduction of MIC values and examine the differences between the MICs of resistant *Candida* isolates against FLZ-NLCs and FLZ-SLNs, and Kruskal–Wallis test was performed for three groups of *Candida* strains (*C. albicans, C. parapsilosis* and *C. glabrata*). P-value less than 0.05 was considered statistically significant. The obtained data were analyzed in SPSS software (version18).

## Results

***Characterization of solid lipid nanoparticles and nanostructured lipid carriers***

**Figure 1:**
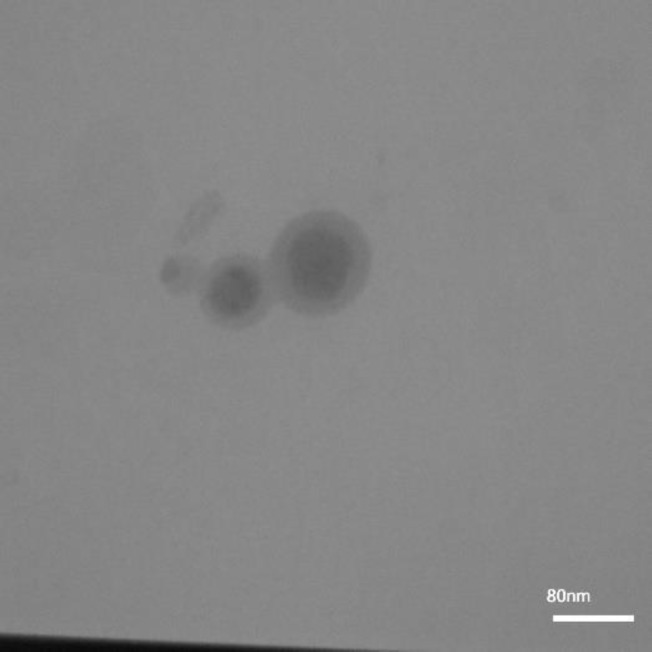
Field emission scanning electron microscope micrograph of fluconazole loaded nanostructure lipid carriers. The particles were observed to be almost spherical in shape with a narrow size distribution

The particle sizes, zeta potential, and entrapment efficiency of the developed SLNs and NLCs are depicted in Table 1. A comparison between FLZ-SLNs and FLZ-NLCs data indicated that the phenomenon for zeta potential can be attributed to the fact that oleic acid is negatively charged at its carboxylic groups; therefore, FLZ-NLCs revealed the highest zeta potential values. Subsequently, the TEM micrographs of both FLZ-SLNs and FLZ-NLCs were suggestive of the spherical nature of the particles (Figure1).

**Table 1:** Component and physicochemical properties of investigated fluconazole loaded solid lipid nanoparticles and fluconazole loaded nanostructure lipid carriers (% w/w)

	**Components**					**Characteristics**			
	**FLZ**	**SA**	**OA**	**Tn80**	**Sn80**	**PS (nm)**	**PI**	**ZP(mV)**	**%EE**
**FLZ-SLN**	0.5	2	-	0.5	0.25	88.5±4.3	0.0290±0.01	-27±2.8	88.3±3.9
**FLZ-NLC**	0.5	1.4	0.6	1.25	2.5	122.6±10.5	0.231±0.01	-36.7±2.5	95.3±3.1

FLZ-SLN: fluconazole loaded solid lipid nanoparticles; FLZ-NLC: fluconazole loaded nonstructured lipid carriers; FLZ: fluconazole

**Table 2:** Effect of new delivery systems for Fluconazole on Fluconazole-resistant strains of *Calbicans*, *C. parapsilosis* and *C. glabrata*

**Isolates**	**Number (n)**	Antifungal agent**	**MIC**													**MIC range**	**MIC50**	**MIC90**	GM*	**Mode**
			**≥64**	**32**	**16**	**8**	**4**	**2**	**1**	**0.5**	**0.25**	**0.125**	**0.062**	**0.031**	**≤0.015**					
*C. albicans*	17 (R)	FLZ	14	-	1	2	-	-	-	-	-	-	-	-	-	8-64	-	-	-	-
		FLZ-NLC	-	-	-	-	-	-	-	-	1	3	5	4	4	0.015-0.25	0.062	0.125	0.059172	0.062
		FLZ-SLN					2	8	3	4						0.5-4	2	4	1.385674	2
*C. glabrata*	13 (R)	FLZ	13	-	-	-	-	-	-	-	-	-	-	-	-	-	-	-	-	-
		FLZ-NLC	--	-	-	-	-	-	-	-	-	2	-	5	6	0.015-0.125	0.015	0.106	0.023019	0.015
		FLZ-SLN					3	6	4							1-4	2	4	1.896155	2
*C. parapsilosis*	9 (R)	FLZ	9	-	-	-	-	-	-	-	-	-	-	-	-	-	-	-	-	-
		FLZ-NLC	-	-	-	-	-	-	-	-	1	-	-	-	8	0.015-0.25	0.015	0.062	0.020505	0.015
FLZ-SLN					2	2	5							1-4	1	4	1.587401	1

* GM: geometric mean MIC; MIC: Minimum Inhibitory Concentrations; FLZ-SLN: fluconazole loaded solid lipid nanoparticles; FLZ-NLC: fluconazole loaded nonstructured lipid carriers; FLZ: fluconazole

***Antifungal susceptibility testing***

Most strains visibly developed after 24 hours of incubation at 35°C using the CLSI document M27-A3 susceptibility testing methodology for FLZ-resistant strains of *C.albicans*, *C. glabrata* and *C. parapsilosis*. However, the results as CLSI recommended were interpreted after 48 h of incubation at 35◦C. Table 2 depicts the results for the in vitro antifungal susceptibility profile of FLZ-SLNs and FLZ-NLCs against tested isolates. A significant decrease was observed in MIC values for strains of *C. albicans*, *C. glabrata* and *C. parapsilosis *after the application of both formulations (*P< 0.05*).Using FLZ-SLNs, the MIC_50_ drug concentration was obtained as 4 µg/ml, all *C. albicans*, *C. parapsilosis* and *C. glabrata* strains. Notably, MIC90 values were obtained as 0.125, 0.062, and 0.106 µg/ml for *C.albicans*, *C. parapsilosis,* and *C. glabrata*, respectively. For all three species, the MICs were significantly decreased with the application of FLZ-NLCs (*P< 0.05*). The comparison of the results obtained for three species groups revealed that the activity of FLZ-NLCs was not statistically significantly different (*P>0.05*). In general, FLZ-NLCs resulted in a significant decrease in MICs (*P<0.05*).

## Discussion

Rare reports have been presented on the resistance to antifungal agents (when compared to antibacterial agents); however, the development of resistance to the current clinically used antifungal agents, especially azole drugs, has emerged as a growing problem [21-23]. Several non-albicans *Candida* species, such as *C. krusei *and newly described *C. auris* strains, are intrinsically resistant or less susceptible to several classes of antifungals, whereas others, including *C. glabrata,* develop acquired resistance following exposure to antifungal agents [23]. Azoles are fungistatic against *Candida* and act by binding to and inhibiting the intracellular target enzyme Erg11p (14-a-demethylase) involved in the biosynthesis of ergostrole. The main mechanisms of resistant against fluconazole include target gene mutation (*ERG11* mutations resulted in less target binding), target up-regulation (*UPC2*, Duplication of chromosome 5 Iso chromosomes), and efflux pumps (*CDR*, *MFS* *CgSNQ2*, *PDH1* (specifically* C. glabrata *[21]) [23]. Therefore, the mechanism that mediates azole resistance is heterogeneous among different species; accordingly, it seems reasonable to assume that lipid core nanocapsules may be able to overcome drug resistance in numerous ways. The present study investigated the effectiveness of two formulations of FLZ-loaded nanoparticles (i.e., FLZ-SLNs and FLZ-NLCs).The nanoparticles with spherical shape and small size were synthesized with excellent zeta potential and entrapment efficiency. There is no question that increasing percentages of oleic acid promotes the drug entrapment efficiency [24]. This observation is in line with the result revealing that liquid lipids, when incorporated with solid lipids, offers enough space to accommodate drug molecules which results in improved drug entrapment efficiency [25]. Another factor that can be attributed to the higher solubility of FLZ in liquid lipid is the improvement of entrapment efficiency. Drug release studies are suggestive of a burst followed by a sustained release behavior which can be associated with the distribution of une-ncapsulated drug on the nanoparticle’s surface and thereafter from the core. Homogeneously distribution of FLZ in the nanoparticles can be a feasible possible justification for the steady release behavior in the initial stage. 

As the results of the current study indicated, FLZ-NLCs could reverse the azole-resistance phenomenon in the most common *Candida* species more effectively, as compared to FLZ-SLNs. Nanostructured lipid carriers have been developed to overcome the pitfalls of SLNs. In NLCs, the combination of lipid with solid lipids results in a higher element drug loading which leads to a higher loading capacity for compounds. In addition, NLCs control the release of the drug and increase the chemical stability of the incorporated drugs [26, 27]. As noted earlier, the overexpression of plasma membrane transport proteins that pump azoles out of cells is a frequent mechanism of high-level azole resistance in fungi which reduces the intracellular azole concentrations below the levels at which Erg11p is inhibited [37,38]. Accordingly, a novel formulation is required to provide a suitable shield for antifungal drugs. The effectiveness of the nano-carrier systems for antifungal delivery has been evaluated in several studies [8, 10, 16]. The results of a study conducted by Bianchin et al. revealed that Lipid core nanoparticles can be considered a novel strategy for the reversal of fluconazole resistance in multiple Candida species [28]. Gupta et al. noted the successful use of lipidic nanoparticles for dermal delivery of fluconazole against cutaneous candidiasis [29]. In the current study, FLZ-NLCs provided an effective nano-scaled safeguard that protects the drug from being pumped out by transporter proteins. Hydrophobic surface of FLZ-NLCs may allow the drug to enter the yeast cells more efficiently. Accordingly, the growing tendency in the emergence of azole-resistant fungal isolates sparks the assessment of the new delivery system for other azoles. 

## Conclusion

Fluconazole solid lipid nanoparticles were found to be more effective against *Candida* species, as compared to FLZ-SLNs. This novel delivery system could face *Candida* strains that exhibit low/no susceptibility to the conventional formulation of FLZ. Based on the obtained results, these novel drug formulations may increase the bioavailability and dissolution rate of VRC to enhance the performance of the formulations.

## Author’s contribution

M. M, HR. K and M. S. designed the project and interpreted the data. AH. D. and M. N. performed the tests and revised and wrote the manuscript. In addition, all of the authors approved the final version of the manuscript.

## Conflicts of interest 

The authors declare that they have no conflict of interest regarding the publication of this paper.

## Financial disclosure

This research was financially supported by a grant (No957487) given to Maryam Moazeni by the National Institute for Medical Research Development (NIMAD). The funder had no role in study design, data collection and interpretation, or the decision to submit the work for publication.
